# Vicarious Menstruation Following the Extraction of a Tooth

**Published:** 1858-06

**Authors:** J. Richardson


### VICARIOUS MENSTRUATION FOLLOWING THE EXTRACTION OF A TOOTH.

BY PROF. J. RICHARDSON.

A married lady, æt about 22 years, called some months since complaining of pain in the right anterior superior molar tooth, which I extracted. The operation was followed by no more than the usual hemorrhage; it soon ceased altogether, and the patient left the office.

On the eighth day after the operation, she again called with an expression of great anxiety and alarm, and stated that the place from which the tooth was taken had been bleeding freely for the past twenty-four hours. On examination I found it still discharging blood, having but little tendency to coagulate. She said that from the time of her first visit, there had been no bleeding from the socket until the day previous to her last call. This circumstance taken in connection with the appearance and condition of the patient, conveyed to my mind at once the impression that the existing homorrhages were connected with some menstrual irregularity. Her face was flushed, the eyes injected, there was a sense of fullness and oppression about the head with headache, pain in the back, nervousness, full pulse, &c., symptoms usually characterizing those periodical perturbations of the circulation in vigorous females, consequent upon a suppression of the catamenia.

Upon further inquiry I elicited from her the fact that there had been, as the result of exposure to cold, no appearance of the menstrual flow for the two previous periods, and that, in the natural order of their recurrence, the third should have taken place at the time of the existing homorrhage.

This discharge from the socket of the extracted tooth, it seemed to me, was clearly vicarious and compensating, and its seat the most convenient one selected by nature to relieve the system of a local determination, and thereby restore the circulation to its just balance.

I applied perchloride of iron to the bleeding surface, prescribed an emmenagogue cathartic, and commended her to her medical adviser for further treatment of her case. I have not seen her since.

